# Evidence That Runt Acts as a Counter-Repressor of Groucho During *Drosophila melanogaster* Primary Sex Determination

**DOI:** 10.1534/g3.120.401384

**Published:** 2020-05-26

**Authors:** Sharvani Mahadeveraju, Young-Ho Jung, James W. Erickson

**Affiliations:** Department of Biology, Texas A&M University, 3258 TAMU, College Station, TX 77843

**Keywords:** X-chromosome counting, Genetic Switch, X-signal element, WRPW, WRPY, deadpan, X:A ratio, Genetics of Sex

## Abstract

Runx proteins are bifunctional transcription factors that both repress and activate transcription in animal cells. Typically, Runx proteins work in concert with other transcriptional regulators, including co-activators and co-repressors to mediate their biological effects. In *Drosophila melanogaster* the archetypal Runx protein, Runt, functions in numerous processes including segmentation, neurogenesis and sex determination. During primary sex determination Runt acts as one of four X-linked signal element (XSE) proteins that direct female-specific activation of the establishment promoter (*Pe*) of the master regulatory gene *Sex-lethal (**Sxl*). Successful activation of *SxlPe* requires that the XSE proteins overcome the repressive effects of maternally deposited Groucho (Gro), a potent co-repressor of the Gro/TLE family. Runx proteins, including Runt, contain a C-terminal peptide, VWRPY, known to bind to Gro/TLE proteins to mediate transcriptional repression. We show that Runt’s VWRPY co-repressor-interaction domain is needed for Runt to activate *SxlPe*. Deletion of the Gro-interaction domain eliminates Runt-ability to activate *SxlPe*, whereas replacement with a higher affinity, VWRPW, sequence promotes Runt-mediated transcription. This suggests that Runt may activate *SxlPe* by antagonizing Gro function, a conclusion consistent with earlier findings that Runt is needed for *Sxl* expression only in embryonic regions with high Gro activity. Surprisingly we found that Runt is not required for the initial activation of *SxlPe*. Instead, Runt is needed to keep *SxlPe* active during the subsequent period of high-level *Sxl* transcription suggesting that Runt helps amplify the difference between female and male XSE signals by counter-repressing Gro in female, but not in male, embryos.

Cell fate decisions are commonly made in response to small quantitative differences in signal molecules. Often such signals are rendered only for brief periods during early development but lead to distinct and permanent cell fates. Sex determination in Drosophila is a well-defined example of a cell fate decision where a transient twofold concentration difference in the proteins that define X-chromosome dose leads to the distinct male and female fates (reviewed in (Cline and Meyer 1996; Salz and Erickson 2010)). Four X-linked genes, *scute (**sc**)*, *sisterlessA (**sisA**)*, *unpaired (upd)* and *run*t* (**run**)* comprise the known X-chromosome signal elements or XSEs (Cline 1988; Duffy and Gergen 1991; Sánchez *et al.* 1994; Sefton *et al.* 2000). The XSEs function collectively to ensure that two X-chromosomes leads to the activation of the master regulatory gene *Sex-lethal (**Sxl**)* and thus to the female fate, whereas a single X-chromosome leaves *Sxl* inactive leading to male development (Cline 1988; Erickson and Quintero 2007). The molecular target of the XSEs is the female-specific *Sxl* establishment promoter, *SxlPe* (Keyes *et al.* 1992; Estes *et al.* 1995). In females, *SxlPe* is activated by the two-X dose of XSEs during a 30-40 min period just prior to the onset of cellularization which occurs about 2:10-2:30 hr after fertilization (Barbash and Cline 1995; Erickson and Quintero 2007; Lu *et al.* 2008; Li *et al.* 2011). The *Sxl* protein products produced from the brief pulse of *SxlPe* activity engage a positive autoregulatory pre-mRNA splicing loop that thereafter maintains *Sxl* protein production from the transcripts made by the constitutive *Sxl* maintenance promoter, *SxlPm* (Cline 1984; Bell *et al.* 1988; Keyes *et al.* 1992; Nagengast *et al.* 2003; Gonzalez *et al.* 2008). In male embryos, the one-X dose of XSEs is insufficient to activate *SxlPe*. Consequently, the transcripts from *SxlPm* are spliced by default so as to produce nonfunctional truncated *Sxl* protein.

The four XSE elements are necessary for proper *Sxl* expression but differ in their sensitivities to gene dose and in their molecular effects on *SxlPe* (Cline 1993). The two “strong” XSEs, *sc* and *sisA*, encode transcriptional activators essential for *SxlPe* expression in all parts of the embryo (Torres and Sanchez 1991; Erickson and Cline 1993; Walker *et al.* 2000). The two “weak” XSEs *upd* and *runt* govern *SxlPe* expression in a broad region in the center of XX embryos, but neither gene is needed for expression at the embryonic poles (Duffy and Gergen 1991; Kramer *et al.* 1999; Avila and Erickson 2007). Changes in *sc* and *sisA* gene dose have dramatic effects on *Sxl* expression and consequently on viability (Cline 1988; Cline 1993). Loss of one copy of each of *sc* and *sisA* is strongly female lethal due to the failure to efficiently activate *SxlPe*. Reciprocally, simultaneous duplication of both genes is strongly male-lethal because *SxlPe* is activated in male embryos bearing an extra dose of *sc**^+^* and *sisA**^+^*.

In contrast to *sc* and *sisA*, both *upd* and *runt* are relatively insensitive to changes in gene dose (Duffy and Gergen 1991; Torres and Sanchez 1992; Cline and Meyer 1996; Kramer *et al.* 1999; Sefton *et al.* 2000). Double heterozygotes between *upd* or *runt* and either of the strong XSEs show comparatively modest effects on *Sxl* expression and on female viability. Duplications of *upd^+^* or *run*t^*+*^ have even smaller effects on male viability as the various combinations lead to, at most, only low-level activation of *Sxl* in XY animals. In the case of *runt*, it was only possible to detect a strong effect of *runt* dose in males, after overexpression by microinjection of *runt* mRNA into embryos (Kramer *et al.* 1999).

The *upd* gene encodes a ligand for the JAK-STAT signaling pathway and its effects on *SxlPe* are mediated via the maternally supplied transcription factor Stat92E (Harrison *et al.* 1998; Jinks *et al.* 2000; Sefton *et al.* 2000). Interestingly, active Stat92E is not needed for the initial activation of *SxlPe* but is required instead to keep the promoter active during the period of maximum *SxlPe* expression (Avila and Erickson 2007). Stat92E binds to several defined DNA sites at *SxlPe* and is thought to be a conventional activator of *SxlPe* transcription that augments the functions of earlier acting XSE proteins but its actual mechanism of action is unknown (Jinks *et al.* 2000; Avila and Erickson 2007).

*runt*, encodes the archetypal member of the Runx (Runt-related transcription factor) family of proteins (Duffy and Gergen 1991; Torres and Sanchez 1992). Runx proteins are highly conserved in metazoans and act, depending on the promoter context, as either activators or repressors in a diverse array of biological processes (Walrad *et al.* 2010; Ito *et al.* 2015; Hughes and Woollard 2017; Mevel *et al.* 2019). Runx proteins are defined by the Runt domain, a 128 amino acid conserved DNA binding domain that binds to the consensus binding site ‘YGYGGY’ (reviewed by (Tahirov and Bushweller 2017)), and by the presence of a conserved C-terminal peptide, VWRPY, that binds to co-repressors of the Groucho/TLE family (Aronson *et al.* 1997; Ito 1997; Jennings *et al.* 2006). Other conserved regions of Runx proteins mediate transcriptional activation and repression independent of the Gro-TLE family (Walrad *et al.* 2010). The *runt* gene is best known for its pair-rule function in embryonic patterning, but its initial role in the fly is as an XSE to establish female-specific expression of *Sxl* in somatic sex determination (Duffy and Gergen 1991; Kramer *et al.* 1999).

In this paper we address the mechanism by which *runt* functions to regulate *SxlPe*. We build on the experiments of Kramer *et al.* (Kramer *et al.* 1999) who demonstrated that Runt works directly on *Sxl* rather than through an intermediary gene. Kramer *et al.* (Kramer *et al.* 1999) considered three general mechanisms for how Runt might control *SxlPe*. First, Runt could act as a conventional direct activator, second; it could facilitate the binding of Sc and SisA transcription factor complexes, or third; Runt could act as a “quencher” of negative regulators. Several observations focused our attention on the third possibility, that Runt activates *SxlPe* by antagonizing Groucho-mediated repression of the promoter.

Maternally supplied Groucho (Gro) is a potent co-repressor of *SxlPe* that is recruited to the promoter by DNA binding repressors of the hairy/E(spl) (Hes)-family, including Deadpan (Paroush *et al.* 1994; Fisher *et al.* 1996; Jennings *et al.* 2006; Lu *et al.* 2008). Loss of Gro leads to ectopic activity of *SxlPe* in males and premature expression in females (Lu *et al.* 2008). The first connection between Runt and Gro was the correlation between the region-specific effects of *runt* on *Sxl* and the region-specific regulation of the co-repressor Gro by the Torso RTK-dependent pathway. In precellular embryos, Gro is phosphorylated directly by MAPK at the embryonic poles with phosphorylation reducing the ability of Gro to repress target genes (Cinnamon *et al.* 2008; Helman *et al.* 2011). Suggestively, the regions where Gro is phosphorylated correspond to the areas where *SxlPe* activity does not depend on *runt* (Duffy and Gergen 1991; Kramer *et al.* 1999). This raised the possibility that Runt is needed only in regions where Gro is highly active, a conjecture supported by early experiments showing that ubiquitous activation of Torso (which leads to ubiquitous phosphorylation of Gro (Cinnamon *et al.* 2008; Cinnamon and Paroush 2008; Helman *et al.* 2011) completely bypassed the requirement for *runt* in *Sxl* expression (Duffy and Gergen 1991). Reasoning that if Runt activates *SxlPe* by interfering with Gro, it would most likely do so via its C-terminal VWRPY peptide, we created *runt* transgenes with or without Gro-interacting motifs. We found that deletion of the WRPY sequence eliminated Runt’s ability to activate *SxlPe*, but that Runt’s transcriptional activation function was restored when the higher-affinity WRPW sequence was used. Since Runt’s ability to activate *SxlPe* depends both on the presence of a functional co-repressor-interacting motif, and an intact DNA binding domain, a straightforward interpretation is that Runt activates *SxlPe* by acting as a “counter-repressor” of Gro function (Pinto *et al.* 2015; Vincent *et al.* 2018). We also demonstrate that Runt is needed only after the onset of *Sxl* transcription, suggesting that *runt*, like *upd* and *Stat92E* (Avila and Erickson 2007), functions to maintain *SxlPe* in an active state. We propose a model suggesting how counter-repression by Runt could both explain Runt’s role in *Sxl* regulation and answer the paradoxical question of how a sparingly dose-sensitive XSE can play a central role in X-chromosome signal amplification.

## Materials and Methods

### Fly culture

Flies were grown at 25° on a standard cornmeal and molasses medium. *w^1118^* flies were received from Bloomington stock center. The *w f run*^3^*/Binsinscy*/Dp(*1*;*Y)y^+^mal*^108^*(run*^+^*)* stock was obtained from T.W. Cline. Null *run^3^* mutant embryos were generated from the cross between *w f run*^3^*/Binsinscy* females and *run*^3^*/Yy^+^mal*^108^*(run^+^)* males. All the transgene lines generated were maintained with two copies in *w f run*^3^*/Binsinscy*/ */Yy^+^mal*^108^*(run*^+^*)* background.

### Plasmids, vectors and transformation

The *run*t*-VWRPY^+^* 10,050 bp genomic fragment, was amplified from *w^1118^* fly genomic DNA using Expand Long Template PCR System (Roche) and cloned into pCR II-TOPO TA vector (Invitrogen). An AvrII site was introduced abutting the *runt* stop codon. The fragment ends are defined by primers: 5′-GGAAAAGTGTGTGGAAAACGGTGGA and 5′-GCAACCCAAATGTCTTGTGAAATGAA. The *run*t*-VWRPY^+^* construct was modified to *run*t*-∆WRPY* and *run*t*-WRPW* using PCR to amplification to change the C-terminal amino acids. The entire *runt* coding sequences, including modifications, were introduced into the genomic clone using an AscI site located in the *runt* 5′ UTR and the introduced AvrII site and confirmed by DNA sequencing. All Runt domain mutations: Cys-127-Ser and Lys-199-Ala, Arg-80-Ala, Arg-139-Ala, Arg-142-Ala, Arg-174-Ala, Arg-177-Ala mutants were generated in pCR II-TOPO TA vector using QuikChange site directed mutagenesis kit (Agilent). The wild type and the respective modifications were confirmed by DNA sequencing. All constructs were cloned, using vector derived EcoRI sites, in the pattB transformation vector kindly provided by Johannes Bischof, Basler lab, Zurich. Transgenic injections were carried out by Genetic services Inc. MA. Constructs were inserted into fly genomic attP2 site on the third chromosome by targeted φC31 mediated specific insertion (Venken *et al.* 2006).

### In situ hybridization

Embryos were collected 0 to 3hr 30 min after the egg laying. Fixation of embryos and *in situ* hybridization with whole mount embryos was as described (Lu *et al.* 2008). Embryos are mounted in 70% glycerol in PBS for imaging. Stages of embryo were detected based on number of nuclei, shape of the nuclei, and cellular furrows as outlined (Lu *et al.* 2008). Templates for *in vitro* RNA transcription was made by PCR amplification with a forward primer and a reverse primer along with T3 promoter using genomic DNA from *w^1118^* flies. A Digoxygenin labeled antisense RNA probe was synthesized using *in vitro* transcription kit (MAXISCRIPT T3 kit, Ambion). Probe was detected using anti-Digoxygenin antibody (Roche) that cross react with NBT-BCIP solution staining the embryos. Primers used to *in vitro* templates were: *Sxl* forward 5′-CCCTACGTCGACGGCATTGCAGC-3′, *Sxl* reverse 5′-TAATACGACTCACTATAGG-GAATGACCCAATGGAATCG-3′ and *runt* forward 5′-AACGACGAAAACTACTGCGGCG-3′, *runt* reverse 5′-AATTAACCCTCACTAAAACGGTCACCTTGATGGCTTTGC-3′.

### Data availability

Strains and plasmids are available upon request. The authors affirm that all data necessary for confirming the conclusions of the article are present within the article, figures, and tables.

## Results

### Runt maintains but does not initiate SxlPe expression

**Figure 1:**
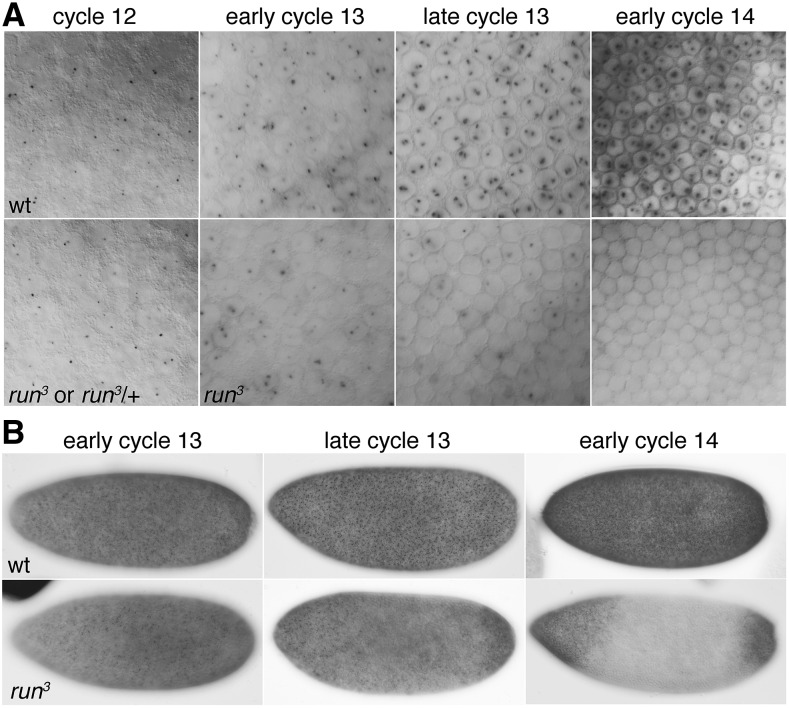
*runt* is needed to maintain but not to initiate *SxlPe* expression. Embryos were stained following *in situ* hybridization to reveal nascent and mature transcripts from *SxlPe*. Dots represent nascent transcripts from the X-linked *SxlPe*. (A) Magnified surface views from the centers of female embryos at the indicated nuclear cycles. Top panel shows wild-type (wt) embryos. Lower panel shows *run^3^* mutant or *run*^3^*/+* embryos. At cycle 12, *run^3^* mutants and *run*^3^*/+* (or wt) could not be distinguished. In cycles 13 and 14 *run^3^* mutants can be unambiguously identified based on staining defects. (B) Whole embryo views of wt or *run^3^* mutant embryos at the indicated nuclear cycles. Embryos are oriented anterior to left, dorsal at top. *run*^3^ null mutants displayed strong *SxlPe* expression at the poles and lowered or no expression in the broad central regions of the embryos. Wild type embryos were *w^1118^*. Mutant embryos from the cross: *w f run*^3^*/Binsinscy* females X *w f run*^3^*/Yy^+^mal*^108^*(run^+^)* males.

Loss of *runt* function eliminates *Sxl* protein and *SxlPe* activity, as measured by *SxlPe-lacZ* transgenes, in a broad central region in early embryos but has no apparent effect on *Sxl* at the anterior and posterior poles (Duffy and Gergen 1991; Kramer *et al.* 1999). To define precisely when and where loss of *runt* affects *SxlPe* we analyzed the effects of the *run^3^* null mutation on the production of nascent transcripts from the endogenous *Sxl* locus. Nascent transcripts from *SxlPe* were visualized as nuclear dots by *in situ* hybridization using an RNA probe derived from the *SxlPe*-specific exon E1 and downstream intron sequences. Typical results are shown in Figure 1 with Figure 1A highlighting nascent transcripts in magnified surface views made from the centers of the whole embryos shown in Figure 1B. As previously reported, *SxlPe*_,_ transcripts appear in wild-type (*w^1118^*) females during nuclear cycle 12 (Erickson and Cline 1998; Avila and Erickson 2007; Erickson and Quintero 2007; Lu *et al.* 2008; Li *et al.* 2011). Initial expression during cycle 12 was mosaic with some nuclei expressing one or both *Sxl* alleles and other nuclei neither allele. By late cycle 12 nearly all nuclei express exhibit two nuclear dots showing that both copies of *SxlPe* are active. This pattern continued, with the dots becoming more intense through cycle 13 and the first 10-15 min of cycle 14 (Figure 1). *SxlPe* activity decreases thereafter with the nuclear dots disappearing by mid cycle 14. Neither wild-type male embryos, nor males carrying a duplication of *run^+^*, express *SxlPe*.

To examine the effects of loss of *runt* function on *SxlPe* activity we examined embryos generated from crosses between *run*^3^*/+* females and *run^3^*/*Yy^+^mal^108^*(*run^+^*) males. Stained progeny were *run*^3^*/ run^3^* and *run*^3^*/+* female embryos in equal proportions. Male embryos with either one or two copies of *run*t^*+*^ do not express *SxlPe* and were unstained. We found over multiple experiments that female embryos completely lacking Runt (*run^3^*/*run^3^*) had obvious defects in *Sxl* expression during cycles 13 and 14. In early cycle 13 *run^3^*/*run^3^* mutants, the defects were evident as a loss of nuclear dots, and thus of expressing nuclei, in the central portions of embryos with the non-expressing regions expanding as cycle 13 progressed (Figure 1). By early cycle 14, *run^3^* null mutants displayed the expression pattern characteristic of *runt* mutants carrying *SxlPe-lacZ* fusions (Duffy and Gergen 1991; Kramer *et al.* 1999): strong expression at the poles and no expression in the broad central regions of the embryos. We note that the *SxlPe* expression phenotype of *run^3^* mutant females was completely penetrant, as one half of the female embryos in cycles 13 and 14 exhibited defective *Sxl* expression patterns (*run*^3^*/run^3^*), whereas their *run*^3^*/+* siblings expressed *SxlPe* in a normal pattern. We could not identify any defects in *SxlPe* expression during nuclear cycle 12 even though one half of the female embryos were *run*^3^*/run^3^* mutants. To confirm that loss of zygotic Runt had no detectable effects on *SxlPe* during cycle 12, we examined all the cycle 12 embryos present in two additional experiments. Out of 47 cycle 12 embryos observed, we found 25 stained female and 22 unstained male embryos. As expected given the mosaic nature of the onset of *SxlPe* expression in cycle 12, the staining patterns of the 25 female embryos were varied, but none appeared outside the norm of wild-type *Sxl* expression, and none exhibited evidence of central region defects as seen in cycle 13 and 14 *run*^3^*/run^3^* females. While these data suggest that Runt is not needed to express *SxlPe* in cycle 12, we cannot exclude the possibility that a low-level of maternally-supplied Runt might promote the initial activation of *SxlPe* in the absence of zygotically provided Runt. We note, however, that *runt* mRNA is not detectable prior to nuclear cycle 10 by RNA-seq in staged single embryos (Lott *et al.* 2011) or by *in situ* hybridization (data not shown).

Our observations suggest that zygotically expressed *runt* is not required for the initial activation of *SxlPe*, but is instead needed to keep the promoter fully active during cycles 13 and 14, but only in the central regions of the embryos. In this sense, *runt* is similar to the XSE *upd* and its associated Stat92E transcription factor, which are likewise dispensable for *SxlPe* activation but required to maintain full *SxlPe* activity after cycle 12 (Avila and Erickson 2007). The “weak” XSE elements are thus both mechanistically distinct from the “strong” XSE activators *sisA* and *scute* that are needed to activate, and presumably to maintain, *SxlPe*, activity in all portions of the embryo.

### Transgenes providing early runt function

**Figure 2:**
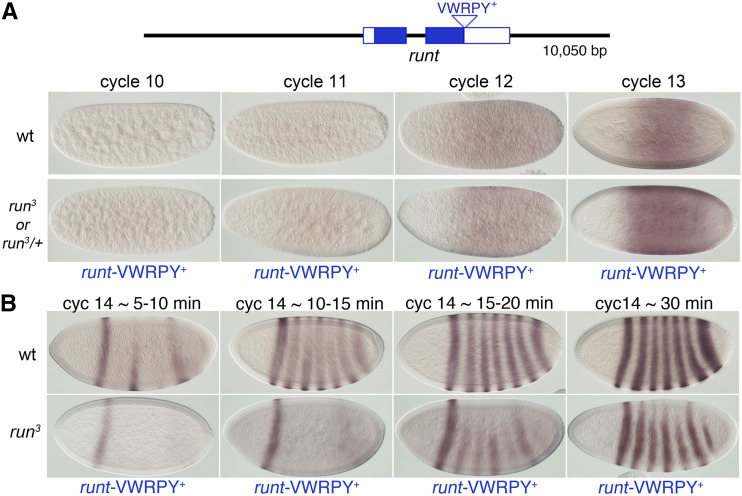
The initial *runt* expression pattern is recapitulated by the *run*t*-VWRPY^+^* transgene, but not the pair-rule expression pattern. Schematic of genomic DNA present in the *run*t*-VWRPY*^+^ transgene. Boxed regions represent coding (solid blue) and non-coding (white) sequences of the *run-RA* transcript (Flybase). The C-terminal peptide VWRPY is indicated. (A) Early *runt* expression pattern. Embryos were stained following *in situ* hybridizations to detect *runt* mRNA. Top panels show wild-type embryos (*w^1118^*) at the indicated nuclear cycles. Lower panels show embryos containing one copy of *run*t*-VWRPY*^+^ from the cross: *w f run*^3^*/Binsinscy* females X *w f run*^3^*/Yy^+^mal*^108^*(run^+^)*; *run*t*-VWRPY^+^* males. Equal numbers of *run^3^* and *run*^3^*/+* females and *+/ Yy^+^mal*^108^*(run*^+^*)* and *run*^3^*/ Yy^+^mal*^108^*(run^+^)* males, each bearing one copy of *run*t*-VWRPY*^+^, were expected. The *run* expression patterns could not be distinguished among the embryo types as all embryos appeared wt. (B) *runt* pair rule expression pattern. Wild type and *run^3^* mutant embryos at the indicated times during nuclear cycle 14 stained to detect *runt* mRNA following *in situ* hybridization. Embryos were staged by nuclear morphology and the degree of cellularization. Stripes are located as in wild type, but are more weakly expressed, particularly in dorsal regions. Embryos are oriented anterior to the left, dorsal to the top. Genetic crosses as in (A).

To further analyze how *runt* regulates *SxlPe* we needed to create transgenes that express *runt* at the proper time and at appropriate levels. The *runt* gene, however, has complex regulatory regions scattered over many kilobases (Butler *et al.* 1992; Klingler *et al.* 1996) and no transgenes have yet been isolated that complement *runt* null mutations. We chose instead to isolate transgenes that reproduced the early *runt* expression pattern needed for its XSE function without concern for all of *run*t*’s* later functions. Using the deletion analysis of Klingler *et al.* (Klingler *et al.* 1996) as a guide we generated a transgene carrying a 10,050 bp genomic fragment, spanning 5,284 bp upstream of the *runt* start codon and 2,824 bp downstream of the *runt* termination codon and integrated it into the 3^rd^ chromosome using site-specific φC31 mediated integration. We named the resulting transgene *run*t*-VWRPY^+^* (Figure 2A). We analyzed the transgenic *runt* expression pattern in the progeny of a cross between *run*^3^*/Binsinscy* females and *run*^3^*/Yy^+^mal*^108^*(run*^+^*)* males carrying the two copies of the *run*t*-VWRPY^+^* transgene. All embryos at or before nuclear cycle 13 expressed *runt* mRNA in patterns indistinguishable from that seen in wild type (Figure 2A). *runt* mRNA was first detectably expressed in nuclear cycle 10. Transcripts gradually increased though cycles 13 without any visible *runt* expression in the anterior. By cycle 13 there was high-level expression in the central regions with greatly reduced mRNA staining in the posterior (Klingler and Gergen 1993). Because we could not distinguish between expression of the *run*t*-VWRPY^+^ t*ransgene and the endogenous *runt* locus in these early embryos in our experiments we examined an additional 100 nuclear cycle 13 embryos, which were predicted to include ∼25 homozygous *run^3^*; *TG/+* females, and observed no deviations from the normal *runt* expression pattern confirming that *run*t*-VWRPY^+^* expresses normally at this stage. While the early *runt* pattern, which is responsible for *run*t*’s* sex determination function (Kramer *et al.* 1999), was expressed normally from the transgenes, one quarter of cycle 14 or older embryos exhibited *runt* staining patterns that differed from the wild-type (Figure 2B) indicating, as expected, that the transgenes lacked some regulatory sequences needed for proper expression of *run*t*’s* segmentation functions.

### Runt-VWRPY^+^ transgenes provide XSE function

**Figure 3:**
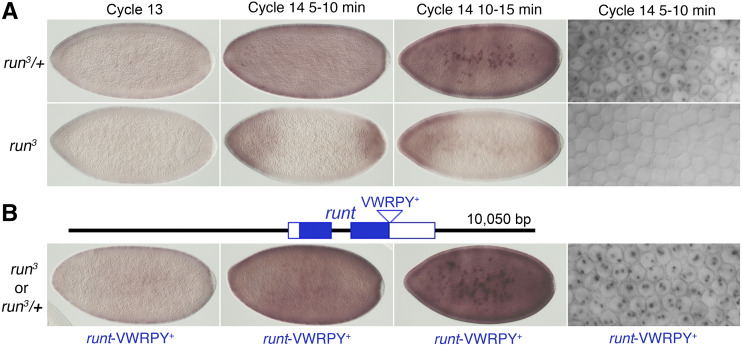
*run*t*-VWRPY^+^* transgene provide full *runt* XSE function. Nascent and mature transcripts from *SxlPe* were visualized after *in situ* hybridization. (A) *SxlPe* expression at the indicated nuclear cycles in *run*^3^*/+* and in *run^3^* mutant females derived from the cross *w f run*^3^*/Binsinscy* X *w f run*^3^*/Yy^+^mal*^108^*(run^+^)*. (B) Schematic depicts the *run*t*-VWRPY^+^* transgene present in single copy in the embryos shown. Since *Sxl* expression appears completely normal in *run^3^* mutants bearing *run*t*-VWRPY^+^* transgenes, we could not determine if the images represent *run^3^* mutants or *run*^3^*/+* heterozygotes. Cross was *w f run*^3^*/Binsinscy* X *w f run*^3^*/Yy^+^mal*^108^*(run^+^)*; *run*t*-VWRPY^+^*.

To determine if the *run*t*-VWRPY^+^* transgene can provide XSE function, we asked if the transgene could restore normal *SxlPe* expression in homozygous *run^3^* mutants. We found that a single copy of the *run*t*-VWRPY^+^* transgene fully complemented the *run^3^* defect as every stained embryo from crosses between *run*^3^*/Binsinscy* females and *run*^3^*/Ymal^108^*; *run*t*-VWRPY^+^* males exhibited a wild-type *SxlPe* staining pattern (Figure 3). Likewise, we could discern no differences in *SxlPe* activity between the *run^3^* mutant and the heterozygous female progeny when the *run*t*-VWRPY^+^* transgene was introduced from the female parents as expected for a zygotically acting XSE (data not shown). Taken together, the complete rescue of *SxlPe* activity in *runt* null mutants and the normal transgenic *runt* expression pattern (Figure 2B) suggest the *run*t*-VWRPY^+^* transgene produces normal or near normal levels of *runt* protein during the time when X chromosome dose is assessed.

### DNA binding is needed for Runt to activate SxlPe

A requirement for Runt DNA binding in *Sxl* activation was reported by Kramer *et al.* (Kramer *et al.* 1999) who found that a *runt* variant carrying two amino acid changes, C127S and K199A (CK), predicted to disrupt DNA binding without greatly perturbing Runt structure, was unable to activate *Sxl* when overexpressed after microinjection of *runt* mRNA into embryos. To confirm that this finding applied to more normal levels of Runt, and to guard against the possibility that the CK amino acid replacements might otherwise alter Runt structure, we introduced the same C127S and K199A changes, as well as five single amino changes (R80A, R139A, R142A, R174A, R177A) predicted to inhibit DNA binding without altering structure (Nagata and Werner 2001) into our *run*t*-VWRPY^+^* transgenes creating *run*t*(DB^m^)* transgenic lines (see Materials and Methods).

We found that each of the amino acid changes abolished the ability of the Runt transgenes to activate *SxlPe* as the female progeny of crosses between *run*^3^*/Binsinscy* females and *run*^3^*/Yy^+^mal^108^*; *run*t*(DB^m^)* males exhibited either the characteristic *run^3^* mutant *SxlPe* staining pattern or the fully wild-type pattern seen in *run*^3^*/+* heterozygotes in the expected 1:1 ratio (data not shown). In no case did we observe evidence for partial complementation confirming that Runt’s DNA binding motif is needed for its XSE function.

### Loss of Runt’s VWRPY Gro-interaction motif abolishes SxlPe expression

**Figure 4:**
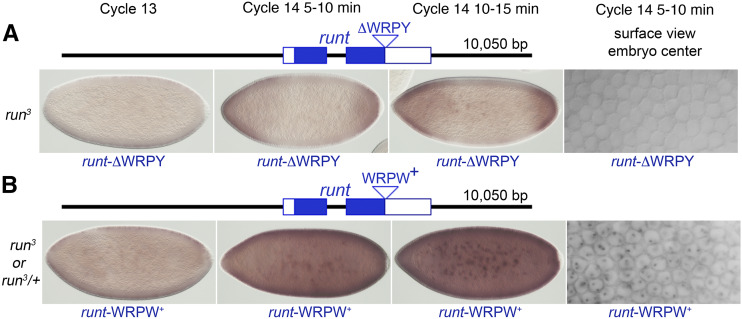
Gro-interacting C-terminal peptides are needed for Runt to activate *SxlPe*. Embryos were stained after *in situ* hybridization to reveal nascent and mature transcripts from *SxlPe*. (A) The Gro-interacting VWRPY peptide is needed for *SxlPe* activation. Schematic shows *run*t*-ΔWRPY* transgene lacking the 4 C-terminal amino acids of the Gro-interacting sequence that is carried in single copy in the embryos shown. Embryos derived from the cross: *w f run*^3^*/Binsinscy* X *w f run*^3^*/ Yy^+^mal108(run^+^)*; *run*t*-ΔWRPY*. (B) Runt protein with the high-affinity Gro binding residues, WRPW, activates *SxlPe*. Schematic shows *run*t*-WRPW* transgene with the Hes protein-derived WRPW Gro-interacting residues carried in single copy in the embryos shown. Embryos were from the cross: *w f run*^3^*/Binsinscy* X *w f run*^3^*/ Yy^+^mal*^108^*(run^+^)*; *run*t*-WRPW*. Since *Sxl* expression appears normal in *run^3^* mutants bearing *run*t*-WRPW* transgenes, we cannot determine if the embryos shown are *run^3^* mutants or *run*^3^*/+* heterozygotes.

To test the significance of Runt’s Gro interactive motif in *SxlPe* activation, the WRPY portion of the motif was precisely deleted from the transgene to produce a *run*t*-∆WRPY* derivative. (Figure 4A). Using φC31-mediated integration, the *run*t*-∆WRPY* transgene was inserted in the same genomic location as the wild type *run*t*-WRPY^+^* transgene. We found that Runt lacking its WRPY motif failed to rescue *SxlPe* expression in *run^3^* mutants (Figure 4A) as one half of the cycle 13 and 14 female progeny of crosses between *run*^3^*/Binsinscy* females and *run*^3^*/Yy^+^mal^108^*; *run*t*-∆WRPY* males exhibited defective *SxlPe* staining patterns indistinguishable from those of *run^3^* mutants alone. Indeed, the *SxlPe* pattern in *run*t*-∆WRPY* bearing *run^3^* null mutants was indistinguishable from *run^3^* mutants alone suggesting that the Gro-interacting WRPY motif is essential for Runt to function as a transcriptional activator at *SxlPe*.

To ensure that the failure of the *run*t*-∆WRPY* transgene to provide sex determination reflected the loss of the WRPY motif, rather than a lack of *runt* protein, we sought a functional assay that would demonstrate the ability of the modified Runt to function in embryos in the absence of the WRPY motif. We chose to examine *fushi tarazu* (*ftz*) as previous work has shown that transcription of *ftz* is partially dependent upon *runt* activity in precellular embryos (Tsai and Gergen 1994; Aronson *et al.* 1997; Swantek and Gergen 2004; Vanderzwan-Butler *et al.* 2007). Most important, *ftz* is activated by Runt in a partially WRPY-independent manner, as overexpressed Runt lacking the C-terminal Gro interaction domain, shows a clear activation of *ftz* expression in regions between the normal *ftz* stripes (Aronson *et al.* 1997).

**Figure 5:**
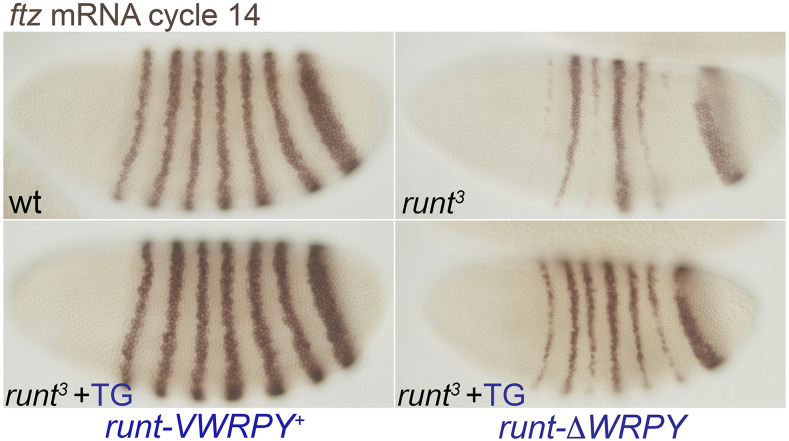
*run*t*-ΔWRPY* transgene retains function as it partially restores *ftz* expression in *run^3^* null mutants. Nuclear cycle 14 embryos stained following *in situ* hybridization to detect *ftz* mRNA. Top panels show *ftz* expression in wild-type and *run^3^* mutant embryos. Lower panels show *run^3^* mutants bearing one copy of *run*t*-VWRPY^+^* or *run*t*-ΔWRPY* transgenes. Crosses were of the form: *w f run*^3^*/Binsinscy* X *w f run*^3^*/ Yy^+^mal*^108^*(run^+^)*; *runt*-transgene.

We first confirmed that expression of *ftz* stripes is reduced prior to gastrulation in *run*^3^ null mutants (Figure 5). We then showed that wild type *run*t*-VWRPY^+^* transgene largely restored the endogenous *ftz* pattern. Critically, we found that the *run*t*-∆WRPY* transgene also restored much of the normal *ftz* pattern in *run^3^* mutants, showing that the *run*t*-∆WRPY* transgene produces functional Runt protein (Figure 5). We note that wild type Runt was more effective at rescuing *ftz* expression than the ∆WRPY derivative. This observation, however, is entirely consistent with previous findings showing that a Runt variant lacking the C-terminal RPY residues was less effective at *ftz* activation than was the wild type when overexpressed (Aronson *et al.* 1997) as well as with the notion that *runt* likely regulates *ftz* expression by more than one mechanism (Aronson *et al.* 1997; Swantek and Gergen 2004; Vanderzwan-Butler *et al.* 2007).

### The potent Gro-interacting motif ‘WRPW’ also provides activation function at SxlPe

Deletion of the WRPY tetrapeptide eliminates both Runt’s interactions with Groucho (Aronson *et al.* 1997) and with its ability to activate *SxlPe* (Figure 3B). We reasoned that if Runt normally employs its VWRPY motif to antagonize Gro-mediated repression at *SxlPe* then it should be possible to substitute a different Gro interaction motif and retain Runt’s ability to activate transcription from *SxlPe*. We chose to test the well-known and potent “WRPW” Gro-interacting motif found in the dedicated repressor proteins of the *hairy-E(spl)* (HES) family. HES proteins bind Gro through their C-terminal ‘WRPW’ motif and recruit it to target gene promoters (Fisher *et al.* 1996; Fisher and Caudy 1998). The molecular interactions of Gro with WRPY and WRPW peptides are similar except that the WRPW peptide interacts with considerably higher affinity (Aronson *et al.* 1997; Jennings *et al.* 2006). We created a *run*t*-WRPW^+^* transgene by changing the C-terminal ‘Y’ residue into ‘W’ and inserted the transgene into the same genomic site as the other transgenes we tested. *In situ* hybridization experiments confirmed that the *run*t*-WRPW^+^* transgene restored normal *SxlPe* expression to female *run^3^* embryos (Figure 4B). This confirms that Runt can act as transcriptional activator of *SxlPe* if its C terminus contains either a VWRPY or VWRPW co-repressor interaction motif.

## Discussion

Drosophila primary sex determination is known for its sensitivity to the concentrations of XSEs and for the rapidity of its response to the sex determination signal. During a 30-40 min period from cycle 12 through early cycle 14, *SxlPe* is turned on, its expression ramped up, and then shut down in female embryos, all while being left inactive in male embryos (Barbash and Cline 1995; Avila and Erickson 2007; Gonzalez *et al.* 2008; Lu *et al.* 2008; Li *et al.* 2011). Despite the short time available, the XSEs appear to act in at least two mechanistic stages: an initiation phase in which X dose is first sensed and a second, maintenance phase, during which the *SxlPe* activity is reinforced (Avila and Erickson 2007). The highly dose-sensitive “strong” XSE proteins, Sc and SisA, appear to act in both stages as complete loss of either, or a twofold reduction in both, effectively eliminate *SxlPe* activity and the temperature-sensitive period for *sc* extends into cellularization (Erickson and Cline 1993; Walker *et al.* 2000; Wrischnik *et al.* 2003). Remarkably the two more weakly dose-sensitive XSE proteins, Runt and Upd, act at the second stage as both are dispensable for the initial activation of *SxlPe* but are critical for maintaining full promoter activity during cycles 13 and 14 (Figure 1, (Avila and Erickson 2007)). A two-step model offers a possible explanation for the paradoxical notion that two critical players in this textbook example of a dose-sensitive genetic switch are themselves relatively dose-insensitive (Duffy and Gergen 1991; Torres and Sanchez 1992; Cline 1993; Sánchez *et al.* 1994; Kramer *et al.* 1999; Sefton *et al.* 2000). The exact gene dose of the weak XSE elements would not matter to male embryos if Runt and Upd, or the Stat92E transcription factor it activates, are only capable of enhancing transcription from an already active *SxlPe*. This could be the case if Runt or Stat92E are unable to bind to or function at *SxlPe* unless the promoter has already been activated by the strong XSE proteins. We note that male-specific viability is unaffected even with a total of four copies of wild-type *runt*, (one each on the *X* and *Yy^+^mal^108^* chromosomes, and two transgenic copies, unpublished data), a finding in stark contrast to what was seen with *sc* or *sisA* which are strongly male-lethal if either one is present in three copies (Erickson and Cline 1991; Erickson and Cline 1993; Cline and Meyer 1996; Wrischnik *et al.* 2003). In females, Runt plays a critical role in maintaining *SxlPe* in the on state during nuclear cycles 13 and 14; however, females would be relatively insensitive to *runt* and *upd* dose if a single copy of each gene provided enough Runt or active Stat92E to effectively reinforce the actions of Sc and SisA. In contrast, if *SxlPe* activity were partially compromised by reductions in *sc* or *sisA* dose an additional reduction in *runt* dose might exacerbate the *Sxl* expression defect leading to the observed female-lethal effects (Duffy and Gergen 1991; Torres and Sanchez 1992).

Evaluating the validity of models of dose-sensitivity requires that the molecular functions of the XSEs be elucidated. The XSE protein Sc and its maternally supplied partner, Daughterless, are bHLH transcriptional activators that bind as heterodimers to six or more sites at *SxlPe* known to be important for transcription (Yang *et al.* 2001). SisA remains an enigma but appears to be a non-canonical bZIP transcription factor (Erickson and Cline 1993; Fassler *et al.* 2002). The *upd* protein signals activation of Stat92E, a maternal transcription factor that binds sequences needed for full *SxlPe* activity (Jinks *et al.* 2000; Avila and Erickson 2007; Cline *et al.* 2010). Stat proteins, like Runx proteins, tend to be relatively weak activators that require interactions with other proteins to activate transcription (Horvath 2000; Goenka and Kaplan 2011). Intriguingly, Stat92E, has been shown to function as a positive regulator of the *crumbs* enhancer, *crb518*, via a counter-repression mechanism (Pinto *et al.* 2015), raising the possibility that Stat92E could function at *SxlPe* in a manner conceptually similar to what we propose here for Runt.

Runt is a bifunctional transcription factor that activates or represses a variety of cellular targets. A common mechanism of repression involves Runt’s C-terminal pentapeptide, VWRPY, which is needed to recruit the potent co-repressor Gro to targets including *even-skipped*, *hairy*, and *engrailed* (Aronson *et al.* 1997; Walrad *et al.* 2010). Still other targets of Runt and Runx proteins are repressed via Gro- and VWRPY-independent mechanisms (Walrad *et al.* 2010; Walrad *et al.* 2011; Hang and Gergen 2017). Activation by Runt is best understood at *sloppy-paired-1* (*slp1*) where Runt interacts with the transcription factor Opa to bind the *slp1* DESE enhancer to drive expression in odd numbered *slp1* stripes (Swantek and Gergen 2004; Walrad *et al.* 2010; Walrad *et al.* 2011; Hang and Gergen 2017). Interestingly, deletion of Runt’s C-terminal 25 amino acids, including the VWRPY motif, prevents Runt from activating *slp1*; however amino acids other than the VWRPY motif appear to be involved as Gro appears to play no role in regulating the DESE enhancer. Here we show that Runt’s ability to promote *SxlPe* activation depends on it possessing a functional Gro-interacting peptide. First, we showed that deletion of just the Gro-interacting WRPY sequence rendered a *runt* transgene that normally provides full XSE function, unable to activate *SxlPe* (Figure 4A). Second, we found that a *runt* derivative containing the higher affinity Gro-interaction motif WRPW sequence from Hes-class repressors also functions as an activator of *SxlPe* (Figure 4B). Critical to our analysis, was the finding that the *run*t*-∆WRPY* transgene that failed to activate *SxlPe* was capable of partially rescuing the *runt*-dependent loss of *ftz* stripes (Figure 5), a function known to be partially dependent on Runt’s WRPY motif (Aronson *et al.* 1997). We attempted to obtain additional evidence for the presence of the Runt-∆WRPY protein in embryos using whole mount immunostaining but were unable to obtain antibody preparations that could detect wild type Runt protein. We acknowledge this limitation of our experiments, but note that deletion of a short C-terminal sequence that included the VWRPY motif did not destabilize Runt when overexpressed in Drosophila salivary glands or early embryos (Walrad *et al.* 2010). Similarly, loss of the VWRPY peptide does not destabilize mammalian Runx1 or Runx3 VWRPY mutants in cultured cells or live animals (Nishimura *et al.* 2004; Yarmus *et al.* 2006; Seo *et al.* 2012).

Our finding that Runt requires its co-repressor interaction domain to function as an activator of *SxlPe* may appear surprising; however, it is not a novel idea. The notion that Runt might act by inhibiting Gro function and act, in current terminology, as a counter-repressor (Pinto *et al.* 2015; Vincent *et al.* 2018), was first raised in the paper that showed the physical interactions between Runt and Gro (Aronson *et al.* 1997). The idea was discussed further by Kramer *et al.* (Kramer *et al.* 1999) and McLarren *et al.* (Mclarren *et al.* 2000; Mclarren *et al.* 2001) who proposed that Runt might activate *SxlPe* transcription by interfering with Gro’s interactions with the Hes-family repressor, Dpn. While we cannot exclude the possibility that Runt’s VWRPY peptide could mediate transcriptional activation via unidentified co-activators, the idea that Runt might antagonize Gro fits well with both the central role of Gro-mediated repression in *SxlPe* regulation (Paroush *et al.* 1994; Lu *et al.* 2008) and with a variety of published data on Gro and Runt function.

Maternally supplied Gro is recruited to *SxlPe* by DNA binding proteins including the Hes protein, Dpn. Dpn binds to three sites within 160 bp of the start of *SxlPe* transcription (Lu *et al.* 2008). While Gro is often considered a long-range repressor, recent analyses have revealed that short-range repression, with Gro-binding near the promoter, as occurs at *SxlPe*, is more common (Kaul *et al.* 2014; Kaul *et al.* 2015). Loss of maternal Gro has several effects on *SxlPe*. It causes ectopic expression in male embryos and premature *SxlPe* activity in females. This suggests maternal Gro defines the initial threshold XSE concentrations needed to activate *SxlPe* and that it actively keeps the promoter off in males. In the absence of Gro, *SxlPe* appears to be expressed in direct proportion to X chromosome dose suggesting that Gro plays a central role in X-signal amplification (Lu *et al.* 2008). Antagonism of Gro function is thus a plausible means by which an XSE might regulate the *SxlPe* switch. The most suggestive prior indication that Runt might work by inhibiting Gro function was that Runt is needed for *Sxl* expression only in the broad central domain of the embryo where Gro-mediated repression is most effective. Runt is not required at the embryonic poles where Torso-signaling leads to the down regulation of Gro activity via phosphorylation (Cinnamon *et al.* 2008; Kaul *et al.* 2015). In this context, the then mysterious observation by Duffy and Gergen (Duffy and Gergen 1991), that a *torso* gain-of-function allele completely bypasses the need for *runt* in *Sxl* activation, is easily explained. Expression of constitutively active torso leads to uniform phosphorylation and inactivation of Gro (Cinnamon *et al.* 2008; Cinnamon and Paroush 2008; Helman *et al.* 2011). Absent active Gro, there is nothing for Runt to counter-repress at *SxlPe*.

How might Runt inhibit Gro function? Based on our findings and those of Kramer *et al.* (Kramer *et al.* 1999) it would appear that Runt must bind to DNA to activate *SxlPe* suggesting that Runt likely inhibits Gro at the promoter. This would rule out a titration scheme in which Runt binds Gro and prevents it from being recruited to *SxlPe* by DNA binding repressors. Plausible mechanisms of Gro inhibition could involve local phosphorylation of Gro at *SxlPe* if Runt could recruit a protein kinase to the promoter, or direct competition with the Hes-repressors, such as Dpn, for Gro-binding (Mclarren *et al.* 2000; Mclarren *et al.* 2001). It is also possible that changes in Gro structure induced by Runt binding to it at *SxlPe* might inactivate Gro. An intriguing possibility is that Runt’s interaction with Gro at *SxlPe* could be mediated by an XSE or an XSE-dependent co-factor. The ability of the Drosophila Runx protein, Lozenge, to stably associate with Gro in eye development depends on its interactions with the transcription factor Cut (Canon and Banerjee 2003). While the interaction with Cut regulates Lozenge’s function as a repressor, a similar mechanism could promote a counter-repressing interaction with Gro.

A remaining mystery is where Runt binds at *SxlPe* as no specific Runt DNA binding sites have been identified near the promoter. Kramer *et al.* (Kramer *et al.* 1999) reported that Runt, and its CBF-β DNA binding partner, Brother (Bro), bound several 200-300 bp DNA fragments from the *SxlPe* region; however, binding specificity was tested only by competitive challenge with high-affinity consensus DNA binding sequences. Our laboratory also found that Runt, in combination with Bro (or the other CBF-β, Big-brother) bound a variety of *SxlPe* fragments, but we observed that binding was efficiently competed in every case by low concentrations of non-specific (poly dI-dC) competitor (unpublished data). Given the absence of obvious matches to the Runt binding site consensus at *SxlPe* and the inability to identify specific *in vitro* binding sites, it suggests that Runt may bind to *SxlPe* only in combination with other protein complexes.

**Figure 6:**
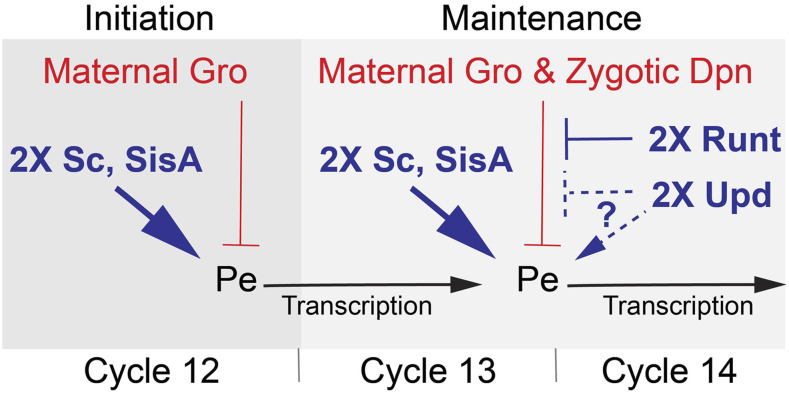
Model for regulation of *SxlPe*. In female embryos the two X dose of the XSE transcription factors, Sc and SisA, overcomes maternal Gro repression initiating expression from *SxlPe* in nuclear cycle 12. During cycles 13 and 14 increased levels of Sc and SisA, assisted by Runt and Upd, maintain *SxlPe* transcription. Runt counter-represses Gro function via its VWRPY domain. Upd, acting through the STAT92E transcription factor, may activate *SxlPe* directly or counteract repression. In male embryos, the single X doses of Sc and SisA fail to overcome Gro-mediated repression and do not activate *SxlPe*. Without *SxlPe* activation, Runt and Upd/Stat92E do not function at *SxlPe*.

The notion that Runt might target Gro function only after *SxlPe* has been activated offers a possible explanation for how the sparingly dose-sensitive, *runt* protein could play an important role in amplifying the twofold difference in male and female XSE doses into a reliable developmental signal. We previously proposed a model in which female-specific dampening of Gro-mediated repression was a central part of X-chromosome signal amplification (Lu *et al.* 2008; Salz and Erickson 2010). Our focus in the earlier paper was a hypothetical feedback mechanism by which active transcription of *Sxl* reduced Gro-mediated repression of *SxlPe*. In the modified version of the model (Figure 6), Runt, and potentially Stat92E, counteract Gro-mediated repression in female, but not in male, embryos. The central tenets of the model are that the 2X dose of the strong XSEs provides sufficient Sc and SisA to cross the threshold for *SxlPe* activation during cycle 12, but that their combined concentrations are insufficient to keep the promoter active in the face of increasing repression as the zygotic repressor Dpn accumulates and translation of maternal Gro mRNA continues. The “weak” XSEs function to counteract repression after *Sxl* transcription begins, either by further enhancing *SxlPe*, as may be the case if Stat92E functions as an activator, or by directly inhibiting Gro function by counter-repression as we propose for Runt. Signal amplification would occur because the increasing XSE protein concentrations in 2X embryos maintains the promoter in an active state, whereas the 1X dose of XSEs can never overcome the ever-increasing repression in males.

Might the kind of counter-repression mechanism we propose for Runt at *Sxl* exist for other genes? Interestingly, McLarren *et al.* (Mclarren *et al.* 2001) observed that mammalian Runx2 inhibited the ability of Hes1 and the mammalian Gro protein, TLE1, to repress an artificial promoter in cultured rat osteosarcoma cells. While the authors did not test if the Runx2 VWRPY residues were needed for relief of TLE1-mediated inhibition, they did note the apparent commonalities with Drosophila sex determination. Further analysis of genes co-regulated by Runx, Hes, and Gro/TLE family proteins should reveal whether it is common for Runx proteins to activate genes by interfering with repression.

## References

[bib1] Aronson B. D., Fisher A. L., Blechman K., Caudy M., and Gergen J. P., 1997 Groucho-dependent and -independent repression activities of Runt domain proteins. Mol. Cell. Biol. 17: 5581–5587. 10.1128/MCB.17.9.5581 9271433 PMC232406

[bib2] Avila F. W., and Erickson J. W., 2007 Drosophila JAK/STAT pathway reveals distinct initiation and reinforcement steps in early transcription of Sxl. Curr. Biol. 17: 643–648. 10.1016/j.cub.2007.02.038 17363251

[bib3] Barbash D. A., and Cline T. W., 1995 Genetic and molecular analysis of the autosomal component of the primary sex determination signal of *Drosophila melanogaster*. Genetics 141: 1451–1471. 8601486 10.1093/genetics/141.4.1451 PMC1206879

[bib4] Bell L. R., Maine E. M., Schedl P., and Cline T. W., 1988 *Sex-lethal*, a *Drosophila* sex determination switch gene, exhibits sex-specific RNA splicing and sequence similarity to RNA binding proteins. Cell 55: 1037–1046. 10.1016/0092-8674(88)90248-6 3144435

[bib5] Butler B. A., Soong J., and Gergen J. P., 1992 The *Drosophila* segmentation gene *runt* has an extended cis-regulatory region that is required for vital expression at other stages of development. Mech. Dev. 39: 17–28. 10.1016/0925-4773(92)90022-C 1336975

[bib6] Canon J., and Banerjee U., 2003 In vivo analysis of a developmental circuit for direct transcriptional activation and repression in the same cell by a Runx protein. Genes Dev. 17: 838–843. 10.1101/gad.1064803 12670867 PMC196027

[bib7] Cinnamon E., Helman A., Ben-Haroush Schyr R., Orian A., Jimenez G., 2008 Multiple RTK pathways downregulate Groucho-mediated repression in Drosophila embryogenesis. Development 135: 829–837. 10.1242/dev.015206 18216172

[bib8] Cinnamon E., and Paroush Z., 2008 Context-dependent regulation of Groucho/TLE-mediated repression. Curr. Opin. Genet. Dev. 18: 435–440. 10.1016/j.gde.2008.07.010 18721877

[bib9] Cline T. W., 1984 Autoregulatory functioning of a *Drosophila* gene product that establishes and maintains the sexually determined state. Genetics 107: 231–277. 6735170 10.1093/genetics/107.2.231 PMC1202321

[bib10] Cline T. W., 1988 Evidence that *sisterless-a* and *sisterless-b* are two of several discrete “numerator elements” of the X:A sex determination signal in *Drosophila* that switch *Sex-lethal* between two alternative stable expression states. Genetics 119: 829–862. 3137120 10.1093/genetics/119.4.829 PMC1203469

[bib11] Cline T. W., 1993 The *Drosophila* sex determination signal: how do flies count to two? [Review] Trends Genet. 9: 385–390. 10.1016/0168-9525(93)90138-8 8310535

[bib12] Cline T. W., Dorsett M., Sun S., Harrison M. M., Dines J., 2010 Evolution of the Drosophila feminizing switch gene Sex-lethal. Genetics 186: 1321–1336. 10.1534/genetics.110.121202 20837995 PMC2998314

[bib13] Cline T. W., and Meyer B. J., 1996 Vive la différence: males vs females in flies vs worms. Annu. Rev. Genet. 30: 637–702. 10.1146/annurev.genet.30.1.637 8982468

[bib14] Duffy J. B., and Gergen J. P., 1991 The *Drosophila* segmentation gene *runt* acts as a position-specific numerator element necessary for the uniform expression of the sex-determining gene *Sex-lethal*. Genes Dev. 5: 2176–2187. 10.1101/gad.5.12a.2176 1748277

[bib15] Erickson J. W., and Cline T. W., 1991 Molecular nature of the *Drosophila* sex determination signal and its link to neurogenesis. Science 251: 1071–1074. 10.1126/science.1900130 1900130

[bib16] Erickson J. W., and Cline T. W., 1993 A bZIP protein, SISTERLESS-A, collaborates with bHLH transcription factors early in *Drosophila* development to determine sex. Genes Dev. 7: 1688–1702. 10.1101/gad.7.9.1688 8370520

[bib17] Erickson J. W., and Cline T. W., 1998 Key aspects of the primary sex determination mechanism are conserved across the genus *Drosophila*. Development 125: 3259–3268. 9671597 10.1242/dev.125.16.3259

[bib18] Erickson J. W., and Quintero J. J., 2007 Indirect Effects of Ploidy Suggest X Chromosome Dose, not the X:A Ratio, Signals Sex in *Drosophila*. PLoS Biol. 5: e332 10.1371/journal.pbio.0050332 18162044 PMC2222971

[bib19] Estes P. A., Keyes L. N., and Schedl P., 1995 Multiple response elements in the *Sex-lethal* early promoter ensure its female-specific expression pattern. Mol. Cell. Biol. 15: 904–917. 10.1128/MCB.15.2.904 7823955 PMC231975

[bib20] Fassler J., Landsman D., Acharya A., Moll J. R., Bonovich M., 2002 B-ZIP proteins encoded by the Drosophila genome: evaluation of potential dimerization partners. Genome Res. 12: 1190–1200. 10.1101/gr.67902 12176927 PMC186634

[bib21] Fisher A. L., and Caudy M., 1998 Groucho proteins: transcriptional corepressors for specific subsets of DNA-binding transcription factors in vertebrates and invertebrates. Genes Dev. 12: 1931–1940. 10.1101/gad.12.13.1931 9649497

[bib22] Fisher A. L., Ohsako S., and Caudy M., 1996 The WRPW motif of the hairy-related basic helix-loop-helix repressor proteins acts as a 4-amino-acid transcription repression and protein-protein interaction domain. Mol. Cell. Biol. 16: 2670–2677. 10.1128/MCB.16.6.2670 8649374 PMC231257

[bib23] Goenka S., and Kaplan M. H., 2011 Transcriptional regulation by STAT6. Immunol. Res. 50: 87–96. 10.1007/s12026-011-8205-2 21442426 PMC3107597

[bib24] Gonzalez A. N., Lu H., and Erickson J. W., 2008 A shared enhancer controls a temporal switch between promoters during Drosophila primary sex determination. Proc. Natl. Acad. Sci. USA 105: 18436–18441. 10.1073/pnas.0805993105 19011108 PMC2587594

[bib25] Hang S., and Gergen J. P., 2017 Different modes of enhancer-specific regulation by Runt and Even-skipped during Drosophila segmentation. Mol. Biol. Cell 28: 681–691. 10.1091/mbc.e16-09-0630 28077616 PMC5328626

[bib26] Harrison D. A., McCoon P. E., Binari R., Gilman M., and Perrimon N., 1998 Drosophila unpaired encodes a secreted protein that activates the JAK signaling pathway. Genes Dev. 12: 3252–3263. 10.1101/gad.12.20.3252 9784499 PMC317220

[bib27] Helman A., Cinnamon E., Mezuman S., Hayouka Z., Von Ohlen T., 2011 Phosphorylation of Groucho mediates RTK feedback inhibition and prolonged pathway target gene expression. Curr. Biol. 21: 1102–1110. 10.1016/j.cub.2011.05.043 21683597

[bib28] Horvath C. M., 2000 STAT proteins and transcriptional responses to extracellular signals. Trends Biochem. Sci. 25: 496–502. 10.1016/S0968-0004(00)01624-8 11050435

[bib29] Hughes S., and Woollard A., 2017 RUNX in Invertebrates. Adv. Exp. Med. Biol. 962: 3–18. 10.1007/978-981-10-3233-2_1 28299647

[bib30] Ito Y., 1997 The runt protein and its companion PEBP2: a close link between this transcription factor and AML. Leukemia 11: 279–280. 9209364

[bib31] Ito Y., Bae S. C., and Chuang L. S., 2015 The RUNX family: developmental regulators in cancer. Nat. Rev. Cancer 15: 81–95. 10.1038/nrc3877 25592647

[bib32] Jennings B. H., Pickles L. M., Wainwright S. M., Roe S. M., Pearl L. H., 2006 Molecular recognition of transcriptional repressor motifs by the WD domain of the Groucho/TLE corepressor. Mol. Cell 22: 645–655. 10.1016/j.molcel.2006.04.024 16762837

[bib33] Jinks T. M., Polydorides A. D., Calhoun G., and Schedl P., 2000 The JAK/STAT signaling pathway is required for the initial choice of sexual identity in Drosophila melanogaster. Mol. Cell 5: 581–587. 10.1016/S1097-2765(00)80451-7 10882142

[bib34] Kaul A., Schuster E., and Jennings B. H., 2014 The Groucho co-repressor is primarily recruited to local target sites in active chromatin to attenuate transcription. PLoS Genet. 10: e1004595 10.1371/journal.pgen.1004595 25165826 PMC4148212

[bib35] Kaul A. K., Schuster E. F., and Jennings B. H., 2015 Recent insights into Groucho co-repressor recruitment and function. Transcription 6: 7–11. 10.1080/21541264.2014.1000709 25603281 PMC4422819

[bib36] Keyes L. N., Cline T. W., and Schedl P., 1992 The primary sex determination signal of Drosophila acts at the level of transcription. Cell 68: 933–943. 10.1016/0092-8674(92)90036-C 1547493

[bib37] Klingler M., and Gergen J. P., 1993 Regulation of runt transcription by Drosophila segmentation genes. Mech. Dev. 43: 3–19. 10.1016/0925-4773(93)90019-T 8240970

[bib38] Klingler M., Soong J., Butler B., and Gergen J. P., 1996 Disperse *vs.* compact elements for the regulation of runt stripes in Drosophila. Dev. Biol. 177: 73–84. 10.1006/dbio.1996.0146 8660878

[bib39] Kramer S. G., Jinks T. M., Schedl P., and Gergen J. P., 1999 Direct activation of *Sex-lethal* transcription by the *Drosophila runt* protein. Development 126: 191–200. 9834198 10.1242/dev.126.1.191

[bib40] Li H., Rodriguez J., Yoo Y., Shareef M. M., Badugu R., 2011 Cooperative and antagonistic contributions of two heterochromatin proteins to transcriptional regulation of the Drosophila sex determination decision. PLoS Genet. 7: e1002122 10.1371/journal.pgen.1002122 21695246 PMC3111545

[bib41] Lott S. E., Villalta J. E., Schroth G. P., Luo S., Tonkin L. A., 2011 Noncanonical compensation of zygotic X transcription in early Drosophila melanogaster development revealed through single-embryo RNA-seq. PLoS Biol. 9: e1000590 10.1371/journal.pbio.1000590 21346796 PMC3035605

[bib42] Lu H., Kozhina E., Mahadevaraju S., Yang D., Avila F. W., 2008 Maternal Groucho and bHLH repressors amplify the dose-sensitive X chromosome signal in Drosophila sex determination. Dev. Biol. 323: 248–260. 10.1016/j.ydbio.2008.08.012 18773886 PMC2653429

[bib43] McLarren K. W., Lo R., Grbavec D., Thirunavukkarasu K., Karsenty G., 2000 The mammalian basic helix loop helix protein HES-1 binds to and modulates the transactivating function of the runt-related factor Cbfa1. J. Biol. Chem. 275: 530–538. 10.1074/jbc.275.1.530 10617648

[bib44] McLarren K. W., Theriault F. M., and Stifani S., 2001 Association with the nuclear matrix and interaction with Groucho and RUNX proteins regulate the transcription repression activity of the basic helix loop helix factor Hes1. J. Biol. Chem. 276: 1578–1584. 10.1074/jbc.M007629200 11035023

[bib45] Mevel R., Draper J. E., Lie-A-Ling M., Kouskoff V., and Lacaud G., 2019 RUNX transcription factors: orchestrators of development. Development 146: dev148296 10.1242/dev.148296 31488508

[bib46] Nagata T., and Werner M. H., 2001 Functional mutagenesis of AML1/RUNX1 and PEBP2 beta/CBF beta define distinct, non-overlapping sites for DNA recognition and heterodimerization by the Runt domain. J. Mol. Biol. 308: 191–203. 10.1006/jmbi.2001.4596 11327761

[bib47] Nagengast A. A., Stitzinger S. M., Tseng C. H., Mount S. M., and Salz H. K., 2003 Sex-lethal splicing autoregulation in vivo: interactions between SEX-LETHAL, the U1 snRNP and U2AF underlie male exon skipping. Development 130: 463–471. 10.1242/dev.00274 12490553

[bib48] Nishimura M., Fukushima-Nakase Y., Fujita Y., Nakao M., Toda S., 2004 VWRPY motif-dependent and -independent roles of AML1/Runx1 transcription factor in murine hematopoietic development. Blood 103: 562–570. 10.1182/blood-2003-06-2109 14504086

[bib49] Paroush Z., Finley R. L. J., Kidd T., Wainwright S. M., Ingham P. W., 1994 Groucho is required for *Drosophila* neurogenesis, segmentation, and sex determination and interacts directly with hairy-related bHLH proteins. Cell 79: 805–815. 10.1016/0092-8674(94)90070-1 8001118

[bib50] Pinto P. B., Espinosa-Vazquez J. M., Rivas M. L., and Hombria J. C., 2015 JAK/STAT and Hox Dynamic Interactions in an Organogenetic Gene Cascade. PLoS Genet. 11: e1005412 10.1371/journal.pgen.1005412 26230388 PMC4521708

[bib51] Salz H. K., and Erickson J. W., 2010 Sex Determination in Drosophila: the view from the top. Fly (Austin) 4: 60–70. 10.4161/fly.4.1.11277 20160499 PMC2855772

[bib52] Sánchez L., Granadino B., and Torres M., 1994 Sex determination in Drosophila melanogaster: X-linked genes involved in the initial step of sex-lethal activation. Dev. Genet. 15: 251–264. 10.1002/dvg.1020150307 8062457

[bib53] Sefton L., Timmer J. R., Zhang Y., Beranger F., and Cline T. W., 2000 An extracellular activator of the Drosophila JAK/STAT pathway is a sex-determination signal element. Nature 405: 970–973. 10.1038/35016119 10879541

[bib54] Seo W., Tanaka H., Miyamoto C., Levanon D., Groner Y., 2012 Roles of VWRPY motif-mediated gene repression by Runx proteins during T-cell development. Immunol. Cell Biol. 90: 827–830. 10.1038/icb.2012.6 22370763

[bib55] Swantek D., and Gergen J. P., 2004 Ftz modulates Runt-dependent activation and repression of segment-polarity gene transcription. Development 131: 2281–2290. 10.1242/dev.01109 15102703

[bib56] Tahirov T. H., and Bushweller J., 2017 Structure and Biophysics of CBFbeta/RUNX and Its Translocation Products. Adv. Exp. Med. Biol. 962: 21–31. 10.1007/978-981-10-3233-2_2 28299648

[bib57] Torres M., and Sanchez L., 1991 The sisterless-b function of the Drosophila gene scute is restricted to the stage when the X:A ratio determines the activity of Sex-lethal. Development 113: 715–722. 1782877 10.1242/dev.113.2.715

[bib58] Torres M., and Sanchez L., 1992 The segmentation gene runt is needed to activate Sex-lethal, a gene that controls sex determination and dosage compensation in Drosophila. Genet. Res. 59: 189–198. 10.1017/S0016672300030470 1511868

[bib59] Tsai C., and Gergen J. P., 1994 Gap gene properties of the pair-rule gene runt during Drosophila segmentation. Development 120: 1671–1683. 8050373 10.1242/dev.120.6.1671

[bib60] Vanderzwan-Butler C. J., Prazak L. M., and Gergen J. P., 2007 The HMG-box protein Lilliputian is required for Runt-dependent activation of the pair-rule gene fushi-tarazu. Dev. Biol. 301: 350–360. 10.1016/j.ydbio.2006.10.027 17137570

[bib61] Venken K. J., He Y., Hoskins R. A., and Bellen H. J., 2006 P[acman]: a BAC transgenic platform for targeted insertion of large DNA fragments in D. melanogaster. Science 314: 1747–1751. 10.1126/science.1134426 17138868

[bib62] Vincent B. J., Staller M. V., Lopez-Rivera F., Bragdon M. D. J., Pym E. C. G., 2018 Hunchback is counter-repressed to regulate even-skipped stripe 2 expression in Drosophila embryos. PLoS Genet. 14: e1007644 10.1371/journal.pgen.1007644 30192762 PMC6145585

[bib63] Walker J. J., Lee K. K., Desai R. N., and Erickson J. W., 2000 The Drosophila melanogaster sex determination gene sisA is required in yolk nuclei for midgut formation. Genetics 155: 191–202. 10790394 10.1093/genetics/155.1.191 PMC1461051

[bib64] Walrad P. B., Hang S., and Gergen J. P., 2011 Hairless is a cofactor for Runt-dependent transcriptional regulation. Mol. Biol. Cell 22: 1364–1374. 10.1091/mbc.e10-06-0483 21325629 PMC3078061

[bib65] Walrad P. B., Hang S., Joseph G. S., Salas J., and Gergen J. P., 2010 Distinct contributions of conserved modules to Runt transcription factor activity. Mol. Biol. Cell 21: 2315–2326. 10.1091/mbc.e09-11-0953 20462957 PMC2893994

[bib66] Wrischnik L. A., Timmer J. R., Megna L. A., and Cline T. W., 2003 Recruitment of the proneural gene scute to the Drosophila sex-determination pathway. Genetics 165: 2007–2027. 14704182 10.1093/genetics/165.4.2007 PMC1462923

[bib67] Yang D., Lu H., Hong Y., Jinks T. M., Estes P. A., 2001 Interpretation of X chromosome dose at Sex-lethal requires non-E-box sites for the basic helix-loop-helix proteins SISB and daughterless. Mol. Cell. Biol. 21: 1581–1592. 10.1128/MCB.21.5.1581-1592.2001 11238895 PMC86704

[bib68] Yarmus M., Woolf E., Bernstein Y., Fainaru O., Negreanu V., 2006 Groucho/transducin-like Enhancer-of-split (TLE)-dependent and -independent transcriptional regulation by Runx3. Proc. Natl. Acad. Sci. USA 103: 7384–7389. 10.1073/pnas.0602470103 16651517 PMC1464349

